# Applicability of a Modified Rat Model of Acute Arthritis for Long-Term Testing of Drug Delivery Systems

**DOI:** 10.3390/pharmaceutics11020070

**Published:** 2019-02-07

**Authors:** Imke Rudnik-Jansen, Nina Woike, Suzanne de Jong, Sabine Versteeg, Marja Kik, Pieter Emans, George Mihov, Jens Thies, Niels Eijkelkamp, Marianna Tryfonidou, Laura Creemers

**Affiliations:** 1 Department of Orthopedics, University Medical Center Utrecht, Heidelberglaan 100, 3508 GA Utrecht, The Netherlands; i.jansen-4@umcutrecht.nl (I.R.-J.); s.e.k.dejong@students.uu.nl (S.d.J.); 2 DSM Biomedical B.V., Koestraat 1, 6167 RA Geleen, The Netherlands; Nina.Woike@dsm.com (N.W.); George.Mihov@dsm.com (G.M.); Jens.Thies@dsm.com (J.T.); 3 Laboratory of Translational Immunology, University Medical Center Utrecht, Utrecht University, Heidelberglaan 100, 3584 CX Utrecht, The Netherlands; sverste2@umcutrecht.nl (S.V.); N.Eijkelkamp@umcutrecht.nl (N.E.); 4 Department of Pathobiology, Faculty of Veterinary Medicine, Utrecht University, Yalelaan 1, 3584 CL Utrecht, The Netherlands; m.kik@uu.nl; 5 Department of Orthopaedic Surgery, Research School CAPHRI, Maastricht University Medical Centre, P. Debyelaan, 25, 6229 HX Maastricht, The Netherlands; pj.emans@maastrichtuniversity.nl; 6 Department of Clinical Sciences of Companion Animals, Faculty of Veterinary Medicine, Utrecht University, Yalelaan 108, 3584 CM Utrecht, The Netherlands; M.A.Tryfonidou@uu.nl

**Keywords:** drug delivery systems, inflammation, arthritis, pain, polyester amide, poly lactic-*co*-glycolic acid

## Abstract

Episodes of inflammation and pain are predominant features of arthritic joint diseases. Drug delivery systems (DDS) could reduce inflammation and pain long-term without chances of infection upon multiple injections. To allow for long-term evaluation of DDS, we modified a previously published acute arthritis model by extending follow-up periods between flare-ups. Unilateral synovial inflammation of the knee was induced by intra-articular injection of streptococcal cell wall peptidoglycan polysaccharide (PGPS), and flare-ups were induced by intravenous PGPS injections every 4 weeks for a total duration of 84 days. In PGPS-reactivated animals, joint swelling, pain behavior, post mortem synovitis, and osteophyte formation were notable features. Hepatitis, splenitis and inflammation of non-primed joints were observed as systemic side effects. To test the applicability of the modified arthritis model for long-term testing of DDS, the duration of anti-inflammatory and analgesic effects of a corticosteroid released from two different polymer-based platforms was evaluated. The current modified arthritis model has good applicability for testing of DDS for a prolonged period of time. Furthermore, the novel autoregulatory polyesteramide (PEA) microsphere platform releasing triamcinolone acetonide (TAA) was benchmarked against poly lactic-*co*-glycolic acid (PLGA) and reduced joint swelling and pain behavior more potently compared to TAA-loaded PLGA microspheres.

## 1. Introduction

Musculoskeletal conditions are the most common cause of severe long-term pain and disability, affecting millions of people worldwide [1]. Musculoskeletal disorders represent a large variety of conditions, of which arthritic diseases of synovial joints are one of the most frequent disorders. Arthritis, or joint inflammation is often used to refer to any disorder that affects the joints and the most representative arthritic diseases are osteoarthritis (OA) and rheumatoid arthritis (RA) [1]. These chronic degenerative joint diseases are characterized by articular cartilage degradation, subchondral bone changes, and recurring inflammation of synovial tissue [2], leading to loss of joint function and reduced mobility among patients. Although the predominant symptom is pain, the exact pain sources and mechanisms are still unclear [2]. Both arthritic diseases present similar joint-associated inflammatory features, in which multiple cartilage degrading proteolytic enzymes induced in the joint in turn have pro-inflammatory roles [2]. Joint pain is generated through pro-inflammatory mediators that sensitize and activate sensory nerves innervating the synovium, or through mechanical stimulation of nociceptors by effusion and/or thickening of the synovium [3,4]. Intra-articular (IA) corticosteroid injection can relieve knee pain and inflammation [5]. However, inhibition of pain generally only lasts 8 to 12 weeks after IA corticosteroid injection [6]. Moreover, multiple corticosteroid injections or their long-term systemic use might entail risks of inducing adverse effects. Biomaterial-based systems for the controlled release of anti-inflammatory drugs might avoid adverse effects by prolonging local drug exposure while decreasing harmful systemic and local peak drug concentrations. Recently, a poly lactic-*co*-glycolic acid (PLGA) microsphere formulation releasing the corticosteroid triamcinolone acetonide (TAA) was developed to prolong inhibition of pain and inflammation in OA knee joints, although the period of pain relief was not different to that of a bolus TAA injection in humans [7,8]. 

Drug delivery systems that prolong anti-inflammatory and/or analgesic effects ideally are tested in vivo over the entire time course of the proposed effects. To address pain as a readout parameter, a rat model of reactivated localized knee arthritis is available. Unilateral synovitis is induced by IA injection of streptococcal cell wall peptidoglycan polysaccharide (PGPS) and flare-up episodes are induced by intravenous PGPS injections every 2 weeks for a total duration of 32 days. However, this model only allows for assessment of treatment efficacy up to day 32. In order to better evaluate the duration of analgesic effects of drug delivery systems as primary objective, we adapted the PGPS model of acute arthritis by extending the periods between each reactivation. Additionally, in this adjusted arthritis model, we investigated the anti-inflammatory and analgesic effects of prolonged TAA release from two different drug delivery systems over 84 days. For this purpose, the recently developed and characterized polyesteramide (PEA) microsphere platform [9,10] releasing TAA was compared to the PLGA microsphere platform already evaluated in the original arthritis model [11,12]. We show here that the modified model of acute arthritis has good applicability for long-term testing of drug delivery systems, but also results in systemic inflammatory features such as hepatitis, splenitis and polyarthritis. Over the course of the prolonged follow-up period, differences in effectiveness of TAA delivery between the PLGA and PEA microsphere platforms were clearly demonstrated as TAA-loaded PEA microspheres were more potent in reducing joint swelling and mechanical hypersensitivity compared to TAA-loaded PLGA microspheres. 

## 2. Materials and Methods 

### 2.1. Loading and Characterization of Microspheres

PEA was synthesized according to a previously published method [13]. PEA polymer was dissolved in dichloromethane (DCM, Merck Millipore, Darmstadt, Germany) and 30 wt% TAA was dispersed in the polymer/solvent mixture. The suspension was sonicated in a water bath for 3 min. Then the formulation was emulsified in 20 mL of water phase, (poly(vinyl alcohol, Sigma-Aldrich, Darmstadt, Germany, 1 wt% and NaCl 2.5 wt%) by the use of an Ultra-Turrax, and stirred at 8000 rpm for 3 min. PLGA microspheres loaded with TAA were synthesized using solid oil-in-water emulsification technique. PLGA (Resomer^®^ RG 753 H, Evonik, Darmstadt, Germany) was dissolved in DCM (Merck Millipore). 25 wt% TAA was dispersed in the polymer/solvent solution and sonicated in a water bath for 3 min to obtain the oil phase. The latter was emulsified in 20 mL of water phase, (poly(vinyl alcohol, Sigma-Aldrich, 1 wt% and NaCl 2.5 wt%) by the use of an Ultra-Turrax and stirred at 4000 rpm for 3 min. PLGA and PEA microspheres were let to harden overnight into a hardening bath of 100 mL water phase under air flow after emulsification. Microspheres were then cooled with an ice-bath for 1 hour and washed with 0.04% Tween 80. Excessive surfactant was removed by centrifugation. Before freeze-drying to remove residuals, particles were resuspended in 0.04% Tween 80. Once dried, closed vials prepared for the in vivo study were sterilized with ɣ-radiation on dry ice. For the in vivo experiment, separate batches of sterilized PEA and PLGA microspheres were used. The batches used in the in vitro experiments were also used and characterized in an unrelated study, including 42 day release profiles (manuscript submitted).

PLGA and PEA microsphere size distribution was measured by static light scattering using a Malvern Mastersizer 2000S. TAA loading was determined by first dissolving 10 mg microspheres in acetone for PLGA microspheres and methanol for PEA microspheres and subsequent diluted in PBS buffer. In vitro release of TAA from the PLGA- and PEA microspheres was determined for 24 weeks (release data up to 42 days were used in another unrelated study). Forty mL of PBS was added to 10 mg PLGA or PEA microspheres and put on a shaker (IKA, IKA^®^-Werke GmbH & Co. KG, Staufen, Germany) at 37 °C for 100 rpm. For high-performance liquid chromatography (HPLC) measurements, vials were first centrifuged for 1 min at 2000 rpm and 36 mL supernatant was removed and replaced with the same amount of PBS buffer. 1 mL supernatant was used for sample analysis using Waters HPLC system. Recovery was performed after the release was stopped and mass balance was determined. Supernatant was removed from the vials and then dried under vacuum at ambient temperature for 48 hours. Afterwards, the white pellet was washed with MilliQ water, centrifuged at 2000 rpm for 2 min and supernatant was removed. This washing step was repeated two times and afterwards, vials were dried again in the oven under the same conditions. The dried microparticles were dissolved in Methanol for PEA and Aceton for PLGA containing samples. Samples were diluted with PBS in such way that they would fit in the range of the calibration curve. Theoretical remaining drug amount was calculated by taking the result of the loading determination and subtracting the cumulative release.

### 2.2. Modified Arthritis Model

#### 2.2.1. Study Design

In this study, 18 female 8 weeks old, Sprague-Dawley rats (Charles-River laboratories, The Netherlands) were used. Six rats served as control group to evaluate the general characteristics of the modified PGPS arthritis model and 12 rats were used to investigate the effects of TAA released by PLGA and PEA microspheres on pain and inflammation inhibition (*n* = 6 per condition). The study design was approved by the National Commission of animal experiments (AVD108002015282) and the working protocol was supervised by the local Animal Welfare Body (WP#800-15-282-01-004) and met the guidelines for animal research in the Netherlands.

Animals were allowed to acclimatize for 7 days prior to the experiments and were housed in groups (3 to 4 rats, randomized) in polycarbonate cages with wire tops, wood chip bedding, and access to ad libitum food and tap water. First, local synovitis was induced (day 28) by priming the experimental knee joint for streptococcal cell wall peptidoglycan polysaccharide (PGPS; 100P fraction with 5 mg rhamnose/mL PGPS from Lee Laboratories) under general isoflurane anesthesia by IA injection of PGPS (25 µL PGPS of 0.17 mg/mL). Flare-up episodes of synovitis were reactivated on day 0, 28, and 56 in the experimental knee joint by injecting PGPS intravenously via the tail vein (0.5 mL PGPS of 0.28 mg/mL). 25 µL PLGA or PEA microspheres releasing TAA were administered 2.5 hours before first reactivation via IA injection in the experimental joint, with total dosage of 2.5 mg/mL TAA. Rats that did not receive any treatment, but were reactivated with PGPS, are referred to ‘untreated rats’ from this point on. Primary experimental outcomes included joint swelling and signs of pain-like behavior (lameness, referred mechanical hypersensitivity) that were measured 0, 1, 2, 4, 15, and 21 days after each reactivation with PGPS. Dynamic weight bearing changes were measured as indication of non-evoked pain-like behavior on day 0, 2 and 15 after PGPS administration. Rescue medication, consisting of 5 mg/kg s.c. carprofen, was given when an animal showed lameness in combination with swelling of both hind paws. In that case, all animals were injected with a single dose of 5 mg/kg carprofen to prevent bias in pain read-out parameters. 12 weeks after TAA delivery, rats were terminated, subsequently scanned with µCT and hind knee joints were collected for histological processing and analyses. All injections and behavioral assays were performed and analyzed in random order by an observer blinded to treatment (IR). Livers and spleens of all rats were macroscopically and microscopically assessed for systemic side effects, by a veterinary pathologist (MK) blinded to treatment.

#### 2.2.2. Joint Swelling

Joint swelling as indicator of inflammation was determined 0, 1, 2, 4, 15, and 21 days after each PGPS administration, by measuring knee joint thickness using a digital caliper. For each time point, joints were measured 3 consecutive times and measurements averaged as one data point. Joint swelling was then calculated by subtracting baseline measurements, performed before priming, from the values of the actual time point.

#### 2.2.3. Referred Mechanical Hypersensitivity

Prior to von Frey measurement, rats were acclimatized for 10 min in a Plexiglas cage with a wire mesh floor. Mechanical sensitivity was assessed by applying von Frey hairs to the hind paw [14]. The 50% threshold was determined using the up-down method, as previously described [15]. In cases where animals showed severe pain behaviors (e.g., curling toes, eversion of the paw, non-weight bearing of parts) continuously for at least 5 min, the lowest value of the von Frey hair was recorded (50% threshold of 0.6 g). 

#### 2.2.4. Dynamic Weight Bearing

The advanced dynamic weight bearing (DWB) device (Bioseb, module version 1.4.2.98; Boulogne, France) was used to measure and analyze weight bearing, as rats with painful experimental joints compensate by redistributing the weight to other weight bearing body parts [16]. For each measurement, each rat was individually placed in a 22 cm × 22 cm × 30 cm plexiglas chamber with floor sensors composed of 44 × 44 captors (10.89 mm^2^ per captor) detecting pressure and a camera that detected the posture of the rat. Rats were allowed to move freely and explore for 5 s prior to subsequent data collection for 5 min. The following parameters were measured: weight on each separate paw (g) and weight on other weight bearing parts (g; front paw, contralateral paw, tail). Three measurements on consecutive days were averaged and used as baseline, thereafter each rat was measured once per time point. Live recordings with a scaled map of the activated sensors was compared to body part placements of the animal. Time spend rearing or washing was excluded from the analyses and a minimum of 1 min of validated testing period was used to calculate mean values. The DWB software (v1.3, Bioseb, Boulogne, France) determined pressure parameters automatically. Zone parameters were set for the analysis as followed: low weight threshold ≥1 g, weight threshold ≥2 g, surface threshold of 3 (in order to be considered a valid zone). For each time segment that was stable for more than one second, zones that met the above criteria were validated and assigned as either right or left and front or hind. A mean value for the weight and area of each zone was calculated over the entire testing period, based on the length of time of each validated segment.

#### 2.2.5. µCT Analyses

Knee joints were imaged post mortem using a Quantum FX µ-CT scanner (PerkinElmer, Waltham, MA, USA) with parameters time = 3 min, isotropic voxel size = 30 µm^3^, tube voltage = 90 kV, and tube current = 180 µA. 3D images were obtained and reconstructed to serial 2D images using software Analyze 11.0 (PerkinElmer, Waltham, MA, USA). Serial 2D or 3D scans of the femur, tibia and patella were made and 2D scans were used to evaluate subchondral sclerosis, osteophytes, bone cysts and loose bodies using ImageJ software (ImageJ, 1.47v, NIH, Bethesda, USA) according to a multi-modality scoring system for rats [17]. 

#### 2.2.6. Histological Processing and Stainings 

All rat cadavers were macroscopically assessed for infectious pathologies, only liver and spleen contained macroscopic pathologies and were further processed for histopathology. Joints were fixed in 4% formaldehyde solution (Klinipath BV, Duiven, Netherlands) at room temperature for 1 week. Thereafter, joints were decalcified at room temperature in Formical 2000 (VWR international BV, Amsterdam, the Netherlands) solution for 4 weeks and embedded in paraffin. Five µm transversal knee joint sections were cut and stained with hematoxylin/eosin to evaluate synovitis using the Krenn score [18] or stained with Safranin-O/Fast green to determine the degree of cartilage degeneration using the Mankin score [19]. Scoring was done at random by an observer blinded for conditions (IR).

#### 2.2.7. Immunohistochemistry 

Immunohistochemistry was performed on 5 µm paraffin sections, to detect pro-inflammatory macrophages indicated as iNOS positive cells and anti-inflammatory macrophages indicated as cd206 positive cells. After deparaffinization and gradual rehydration, antigen retrieval was performed by boiling sections at 70 °C for 30 min in 10 mM citrate buffer with pH = 6. After cooling down of sections, blocking for nonspecific endogenous peroxidase was done for 10 min in 0.3% H_2_O_2_ and slides were subsequently washed twice with PBS containing 0.1% Tween20 (PBST) for 5 min. Then, sections were blocked in PBS containing 5% BSA for 30 min before the overnight incubation at 4 °C with the primary antibodies (0.52 µg/mL Rabbit polyclonal iNOS, Ab15323 from Abcam, with 0.52 µg/mL rabbit IgG isotype as negative control. 3.3 µg/mL cd206 goat polyclonal IgG, AF2535 from R&D systems with 3.3 µg/mL Goat igG isotype as negative control). The next day, sections were washed with PBST before incubation with the secondary antibody for 30 min at room temperature (BrightVision poly-HRP-anti rabbit, K4002 from Dako or 4 µg/mL Donkey anti-Goat igG H&L, ab6886 from Abcam). After washing with PBS, iNOS sections containing HRP-labeled antibodies were incubated with DAB substrate for 5 min, counterstained with Weigert’s Hematoxylin for 3 min and rinsed with running tap water for 10 min. Before permanent mounting with Depex (06522, Sigma-Aldrich, Darmstadt, Germany), sections were dehydrated with series ethanol and xylene. After washing with PBS, cd206 sections containing AP-labeled antibodies were incubated with Ferangi Blue™ chromogen kit 2 (FB813H from Biocare medical), rinsed twice with deionized water, and air dried at 37 °C. Permanent mounting was done using EcoMount (SKU: EM897L from Biocare medical). For quantification of the subsequent iNOS- and cd206 sections, of each joint per microscope slide, three digital images were obtained of the exact same region (medial synovium, lateral synovium, and cruciate ligament) and all iNOS- or cd206 positive cells were manually counted and averaged using Photoshop count tool (Adobe Photoshop CS6, version 12.0.1x64).

### 2.3. Statistical Analyses

All data were analyzed using IBM^®^ SPSS^®^ Statistics version 21. For each dataset, treatment period of 84 days for every animal was used and statistical analysis was performed to detect differences between affected and contralateral joints in untreated animals, or differences in the affected joints between the PEA and PLGA platform. Equality of data variances was evaluated by quantile-quantile plots and homoscedasticity of residuals by scatterplots. In case ANOVA assumptions were not met, the Kruskal–Wallis test was used to analyze nonparametric data. Osteophyte formation and bone cyst number of the affected ipsilateral joints were compared to the contralateral knee joints using the nonparametric Mann–Whitney U test. Read-out parameters weight, ipsilateral synovitis, contralateral synovitis, and iNOS/cd206 cell number ratio of the three groups were analyzed using one-way ANOVA with Bonferroni correction for multiple comparisons in case of statistical differences between subjects. The one-way ANOVA with Bonferroni correction was performed to detect statistical differences in body weight during the immediate period after local TAA injection on day 2. Differences between the PLGA and PEA platform in the course over time for joint swelling and 50% response thresholds were analyzed by nonparametric Kruskal–Wallis test with post-hoc pairwise comparisons using the Dunn–Bonferroni approach. The weight asymmetry over time comparing the PLGA platform to the PEA platform, was analyzed by nonparametric Mann–Whitney U test. Statistical significant differences were found at *p* < 0.05.

## 3. Results

### 3.1. General Observations

#### 3.1.1. Systemic Effects of the PGPS Reactivations

Four rats were euthanized before the end of the study (no treatment *n* = 2 on day 56 and 70, TAA PEA *n* = 2 on day 57 and 70) as the humane endpoint was reached due to polyarthritis unresponsive to rescue pain medication. 

**Figure 1:**
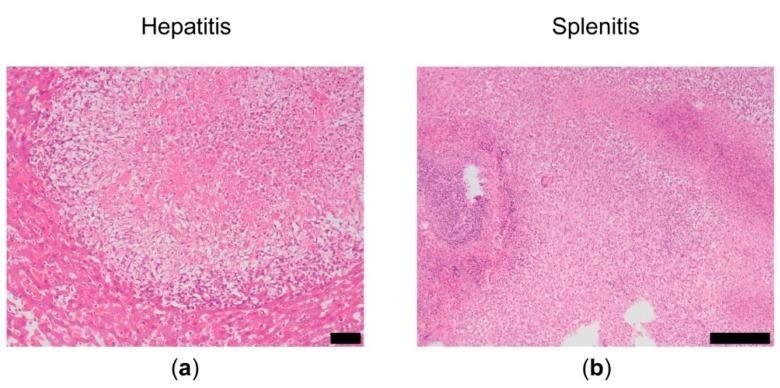
Microscopic overview of (**a**) liver and (**b**) spleen after the initial priming and the three reactivations that indicate signs of hepatitis and splenitis. Scale bar left panel 50 µm and right panel 100 µm.

Polyarthritis occasionally presented as swelling of the contralateral knee and ankles. The average body weight increased from 215.7 g ± 13 g to 336.5 g ± 14 g during the study period. No clinical signs of *streptococcus* infection (e.g., dyspnea, weight loss, hunched posture) [20] were observed. However, macroscopic and microscopic analysis of liver and spleens revealed granulomatous hepatitis and granulomatous splenitis, ranging from a slight reaction to a severe reaction (Figure 1). No abnormalities (scored as no–negligible cellular reactions) were observed in in the liver of 33% of the untreated animals, 50% of animals treated with TAA-loaded PLGA microspheres and 83% of animals treated with TAA-loaded PEA microspheres. In the spleen, no abnormalities were observed in 33% of the untreated animals and 33% of the animals treated with TAA-loaded PEA microspheres (Table 1). No macroscopic abnormalities were observed in the other organs (lungs, heart, intestine, urinary bladder). 

#### 3.1.2. Histological Joint Pathology

**Figure 2:**
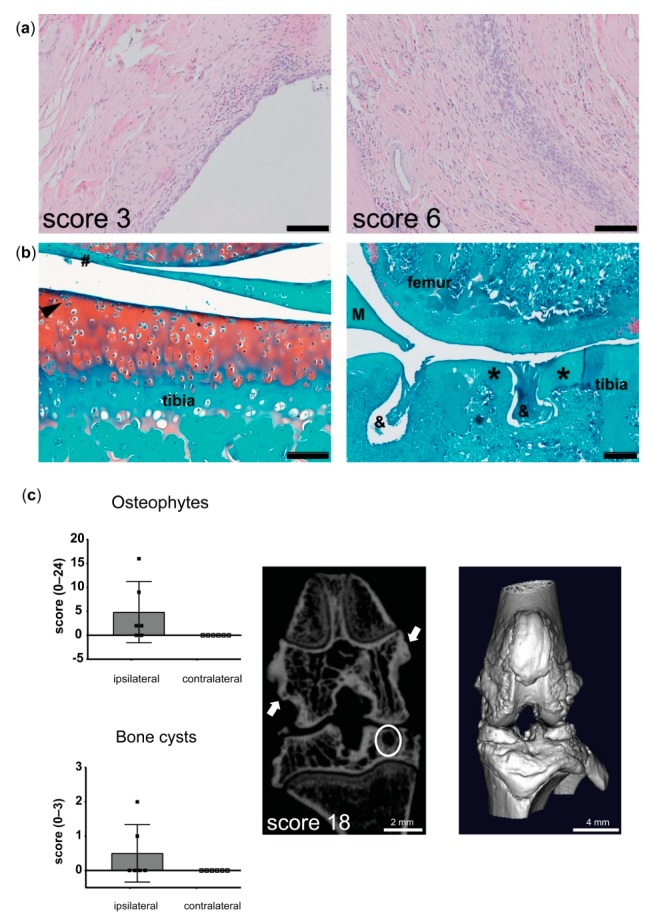
Osteoarthritis (OA)-related features in joints from rats in the modified arthritis model. (**a**) Left; microscopic image of the best outcome of Krenn score, reflecting mild synovitis (3 out of 9), right; microscopic image of the worst outcome of Krenn score, showing moderate–severe synovitis (6 out of 9). Scale bars = 100 µm. (**b**) Left; microscopic image of the best outcome of Mankin score, reflecting very mild cartilage degeneration, hashtag indicates pannus formation, black arrow shows cell cloning. Scale bar = 100 µm. Right; microscopic image of the worst outcome of Mankin score. Asterisk indicates loss of proteoglycans and hypo-cellularity. Ampersand shows bone erosion. M = meniscus. Scale bar = 20 µm. (**c**) Left; quantification of osteophyte and bone cyst formations in the affected ipsilateral and contralateral knee joints of untreated animals that were reactivated with peptidoglycan polysaccharide (PGPS). Data represent mean ± standard deviation (SD). Right; 2D and 3D µCT image of the affected joint from an untreated rat. Arrows indicate osteophytes and a bone cyst in the tibia plateau is encircled.

PGPS-treated and -reactivated affected knee joints displayed activation of synovial stroma with a thickened synovial lining and extensive immune cell infiltration (Figure 2a). Cartilage degeneration was mild (6 out of 14), reflected by mild pannus formation, surface irregularities, cloning of chondrocytes, and a mild loss of proteoglycan staining (Figure 2b). The number of osteophytes and bone cysts in the affected ipsilateral knee joints trended to an increase compared to contralateral joints (Figure 2c). Despite joint swelling in the contralateral knee, signs of synovitis in the contralateral knee joints were mild; score ≤ 2 out of 9 (Figure 2a).

#### 3.1.3. Course of Inflammation, Hyperalgesia and Weight Bearing in Untreated Animals in the Modified Arthritis Model

**Figure 3:**
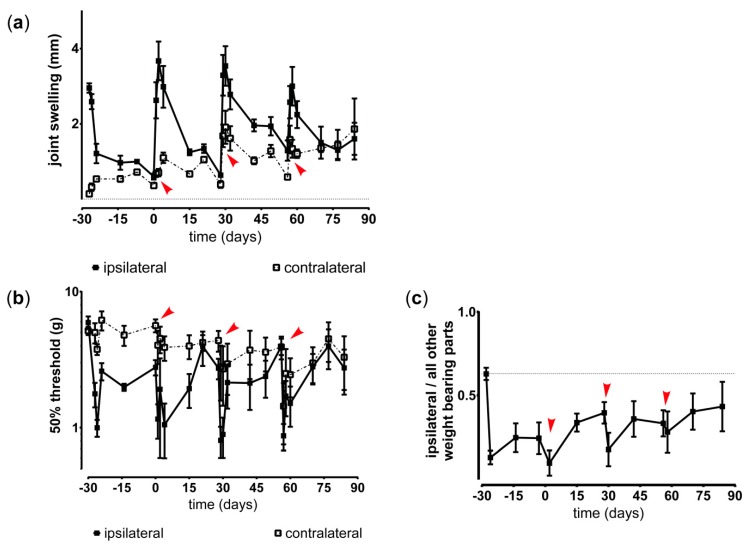
Inflammation and hyperalgesia induced by streptococcal cell wall peptidoglycan polysaccharide (PGPS) reactivations in a modified model enabling long-term monitoring of pain. (**a**) Joint swelling, as indication of inflammation, was measured in untreated affected (ipsilateral) and contralateral (contralateral) knee joints. PGPS reactivations on day 0, 28, and 56 are indicated by red arrows. (**b**) Withdrawal threshold of the induced mechanical hypersensitivity in untreated affected (ipsilateral) and contralateral (contralateral) knee joints. PGPS reactivations on day 0, 28, and 56 are indicated by red arrows. (**c**) Inflammation-induced changes in the ratio of weight bearing of the affected ipsilateral paw compared to the other weight bearing parts in untreated rats, reactivated with PGPS on day 0, 28, and 56, indicated by red arrows. All data represent mean ± standard deviation (SD).

Joint swelling as sign of acute inflammatory responses was induced by PGPS injections and resolved around 14 days post injection (Figure 3a). PGPS priming and reactivations also induced mild joint swelling of the contralateral knee. The inflammatory episodes coincided with increased mechanical hypersensitivity of the affected hind paw (Figure 3b). Weight bearing of the affected hind paw was reduced and did not completely return to baseline before the next reactivation, indicating persistent non-evoked pain behavior (Figure 3c). 

### 3.2. Polymer-Based Drug Delivery

#### 3.2.1. PLGA and PEA Microsphere Characterization

PLGA batches used in the in vitro study showed a mean size of 32 µm with a polydispersity index of 1.344. PEA batches used in the in vitro study showed a mean size of 23.6 µm with a polydispersity index (PDI) of 1.206. TAA loading of these PLGA-loaded microspheres was 22.8 wt% ± 0.01 with a loading efficacy of 91%. TAA loading of these PEA-loaded microspheres was 18 wt% ± 0.01 with a loading efficacy of 101%.

PEA and PLGA batches used for the in vivo study also showed a monomodal size distribution, ranging from 8 to 50 µm. Mean size of PLGA microspheres were 39.4 µm with a PDI of 1.30. Mean size of PEA microspheres was 23.8 µm, with PDI of 1.30. TAA loading of PLGA-loaded microspheres was 23 wt% TAA and for PEA-loaded microspheres 28 wt% TAA. For both formulations the loading efficacy was 92%. 

#### 3.2.2. In Vitro Drug Release Kinetics of PLGA and PEA Microspheres.

**Figure 4:**
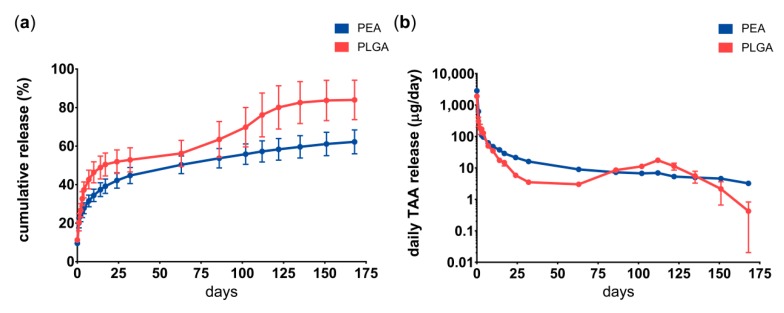
Triamcinolone acetonide from autoregulatory polyesteramide (PEA) or PLGA microspheres is released in vitro over 24 weeks. (**a**) Cumulative triamcinolone acetonide (TAA) release from PLGA (red) or PEA (blue) microspheres in PBS medium (left panel) and (**b**) daily TAA release from PLGA (red) or PEA (blue) microspheres (right panel). Data represent mean ± standard deviation (SD) of three microsphere batches per polymer.

Drug release from PEA microspheres in PBS followed a sustained release after the initial burst during the first 14 days, while drug release from PLGA microspheres showed two burst phases; one during the first 2 weeks and one after 12 weeks (Figure 4). Cumulative TAA release from PLGA microspheres was 80% and from PEA microspheres 60% after 24 weeks. Typically, small amounts of microspheres are lost during buffer exchange, most likely accounting for the approximately 20% TAA lost. Drug recovery at the end point of the study was on average 22% TAA for PEA microspheres and 0% from PLGA microspheres. 

#### 3.2.3. Effects of Locally Delivered TAA 

**Figure 5:**
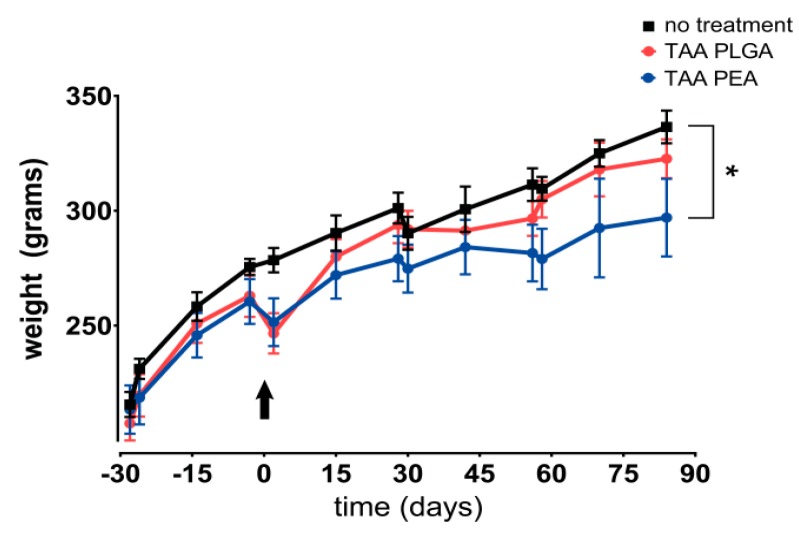
Body weight gain during the 84 day follow-up of the study. Animals are intra-articularly injected with TAA-loaded PLGA microspheres (red) or TAA-loaded PEA microspheres (blue) and compared to untreated animals (black). In the immediate period after local TAA injection, PEA-treated and PLGA-treated animals reduced in 3% and 7% body weight, respectively. Arrow indicates time point (d0) of intra-articular microsphere injections. Data represent mean ± standard deviation (SD), * *p* < 0.05.

The body weight of rats treated with TAA-loaded PEA microspheres increased during the study period, although significantly less than untreated or rats treated with TAA-loaded PLGA microspheres (Figure 5). In the immediate period after local TAA injection, compared to untreated animals, a loss of 3% in PEA-treated animals was observed (*p* = 0.092) and of 7% in PLGA-treated animals (*p* < 0.05). Animals receiving the latter treatment recovered thereafter to similar body weight levels as untreated animals. TAA-loaded PEA microspheres reduced the systemic PGPS-induced hepatitis slightly, while TAA delivery for both polymer-based delivery systems did not affect splenitis (Table 1).

**Figure 6:**
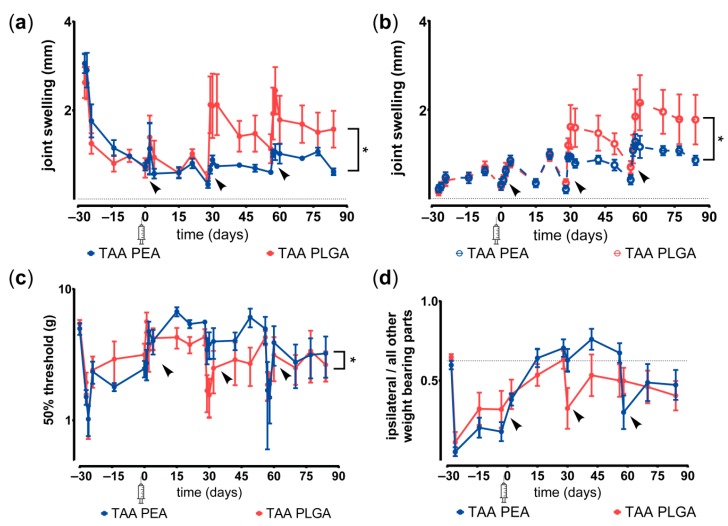
Anti-inflammatory and analgesic effects of TAA-loaded PLGA and PEA microspheres. (**a**) Joint swelling, as sign of inflammation, of the affected knee joints and (**b**) contralateral knee joints after single intra-articular injection of TAA-loaded PLGA (red) or TAA-loaded PEA microspheres (blue). Synovitis reactivations were evoked at day 0, 28, and 56, indicated by black arrows. (**c**) Withdrawal threshold of the affected (ipsilateral) hind paw after single intra-articular injection of TAA-loaded PLGA (red) or TAA-loaded PEA microspheres (blue). Synovitis reactivations were evoked at day 0, 28 and 56, indicated by black arrows. (**d**) Inflammation-induced changes in the ratio of weight bearing of the affected paw compared to the other weight bearing parts of the rat treated with TAA-loaded PLGA microspheres (red) or TAA-loaded PEA microspheres (blue). Synovitis reactivations were evoked at day 0, 28, and 56, indicated by black arrows. Syringe indicates time point of intra-articular microsphere injection. Data represent mean ± standard deviation (SD), * *p* < 0.05.

TAA released from PEA microspheres effectively reduced joint swelling over the entire study period, while the inhibition of joint swelling by TAA-loaded PLGA microspheres declined after the second reactivation in joints (Figure 6a). Mild joint swelling of the contralateral knee joint occurred in animals receiving either microsphere platform, however TAA-loaded PEA microspheres prevented swelling in the contralateral knee more than TAA-loaded PLGA (Figure 6b). TAA-loaded PEA microspheres also reduced mechanical hypersensitivity of the hind paw more effectively than TAA-loaded PLGA microspheres (Figure 6c). Weight bearing deficits of the affected ipsilateral hind paw resolved in rats treated with TAA-loaded PLGA and PEA microspheres until the third reactivation. After the third reactivation weight bearing of the affected joint was reduced again (Figure 6d). The two microsphere platforms did not statistically differ in attenuating the weight distribution deficits when analyzed over the entire study period. 

#### 3.2.4. Effects of TAA Delivery on Synovitis, Macrophage Subtypes and OA-Like Bone Phenotypes 

**Figure 7:**
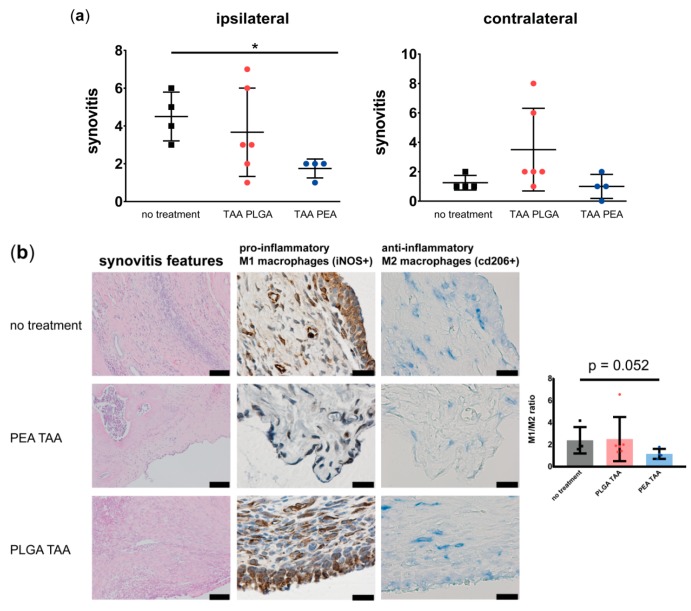
Histology and immunohistochemistry of untreated knee joints compared to joints treated with TAA-loaded PLGA microspheres and PEA microspheres. (**a**) Synovitis of affected knee joint (left panel) and contralateral knee joint (right panel) was quantified by Krenn scoring of knee joints treated with a single intra-articular injection of TAA-loaded PLGA microspheres (red) or TAA-loaded PEA microspheres (blue) and compared with untreated knee joints (no treatment). Data are presented as mean ± standard deviation (SD) and significance with * = *p* < 0.05. (**b**) Worst synovitis outcome visualized by H&E staining (left panel) with scale bars = 100 µm, presence of pro-inflammatory, iNOS positive, macrophages (middle panel) with scale bars = 20 µm, and presence of anti-inflammatory, cd206 positive, macrophages (right panel) with scale bars = 20 µm. Quantified ratio of M1/M2 macrophages is also displayed.

Moderate to mild synovitis was present in knee joints treated with TAA-loaded PLGA- or PEA microspheres. However, only TAA-loaded PEA microspheres significantly reduced the synovitis compared to untreated joints (Figure 7a; left panel). In the contralateral knee joints, signs of synovitis were mild (score ≤ 2 out of 9) in all rats treated with TAA-loaded PEA microspheres, whilst 67% of the TAA-loaded PLGA microsphere treated rats had mild synovitis (Figure 7a; right panel). TAA released by the PLGA or PEA microspheres did not affect cartilage degeneration in ipsilateral nor contralateral joint (Supplementary file 1). A trend was observed towards an increase in anti-inflammatory M2 macrophages in the synovial tissue of TAA-loaded PEA microsphere treated joints, compared to untreated or TAA-loaded PLGA microsphere treated joints (Figure 7b, *p* = 0.052). 

**Figure 8:**
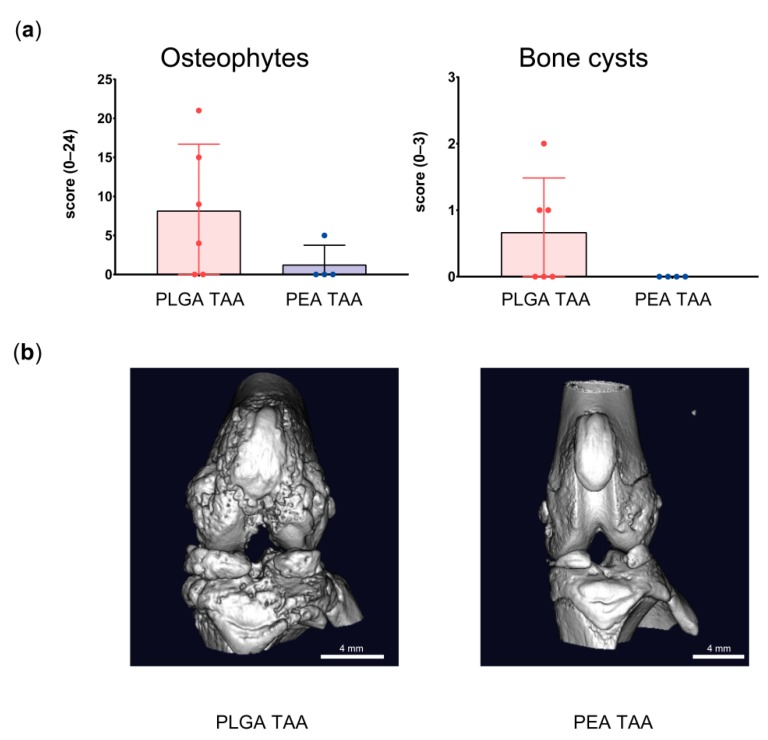
Post mortem imaging of bone changes in joints treated with TAA-loaded PLGA and PEA microspheres. (**a**) Osteophyte and bone cyst formation in the affected ipsilateral knee joints of animals treated with TAA-loaded PLGA microspheres (red) or TAA-loaded PEA microspheres (blue). Data are presented as mean ± standard deviation (SD). (**b**) 3D µCT images of the affected joints treated with TAA-loaded PLGA microspheres (left) and PEA microspheres (right).

Post mortem µ-CT analysis of the affected knee joint showed a trend (*p* < 0.1) towards an inhibition of osteophyte formation and number of bone cysts in TAA-loaded PEA microsphere treated joints compared to TAA-loaded PLGA treated knee joints (Figure 8).

## 4. Discussion

In this study we modified the rat model of PGPS-induced acute arthritis in order to allow for long-term evaluation of drug delivery systems. To this end, inflammatory-induced hyperalgesia was evoked by PGPS injections every 4 weeks instead of a 2-week interval to extend the total follow-up to 84 days. Systemic effects were observed including hepatitis, splenitis and mild polyarthritis. Joint pathology comprised moderate synovitis and very mild articular cartilage degeneration, with osteophyte formation as a more OA-like characteristic. The model was used to evaluate the anti-inflammatory and analgesic effects of two polymer-based drug delivery platforms hypothesized to have a differential long-term drug release based on in vitro release studies and their design characteristics. A temporal loss in body weight was observed immediately after IA delivery of TAA by both platforms. A single intra-articular injection of TAA-loaded PEA microspheres reduced body weight gain over the remaining period, but also prevented the PGPS-induced hepatitis. Other systemic effects of TAA-loaded PLGA microspheres were not detected. PEA microsphere-mediated TAA release reduced swelling and mechanical hypersensitivity to a greater extent compared to TAA released by PLGA microspheres, while compensation in the weight bearing asymmetry was similar for both platforms. Furthermore, a trend towards reduced osteophyte and bone cyst formation in joints treated with TAA-loaded PEA microspheres compared to joints treated with TAA-loaded PLGA microspheres was observed. 

Although this modified model allows for long-term evaluation of drug delivery system, a drawback of the current arthritis model, negatively affecting the power of the study, is that some animals are lost to follow up due to untreatable pain. This was probably unrelated to the reactivation procedures, nor the treatment allocation, although the small sample size impede conformation by statistical analysis. Episodes of acute inflammation recurred spontaneously in between reactivation intervals, and the variability was high, something also observed in RA patients [21,22]. It is not clear to what extent comparable follow-up losses were encountered in the previous studies on the original PGPS rat model of arthritis, they do not report animal wellbeing or loss [11,12]. Here, we evaluated for the first time potential systemic effects occurring in the acute arthritis model. Whether similar systemic effects are specific for this modified arthritis model remains to be determined, since splenitis and hepatitis as part of the systemic inflammation do not appear to have been evaluated before in the studies based on the original version of the model. There are, however, several reports underscoring the systemic inflammation component of models in which bacterial cell wall components are employed. In studies based on a single intraperitoneal injection of PGPS consisting of 60 µg rhamnose / g body weight, cell wall material could be traced in organs in small amounts after 180 days of single intraperitoneal injection, which resulted in histopathological fibrous connective tissue and foci of macrophage accumulation [21,23]. The described pathology was milder compared to the accumulations found in the present study, probably inherent to repetitive reactivation by intravenous PGPS injection rather than the cumulative rhamnose dose of 30 µg rhamnose/g body weight. Also in the lipopolysaccharide (LPS)-induced arthritis model, spleens and livers contained pronounced immune cell infiltrations or apoptotic processes, 24 hours after injection [24]. In combined collagen-induced arthritis accelerated by intraperitoneal LPS injection, ‘ballooning’ of hepatocytes was observed after 28 days [25]. Hence, most likely the splenitis and/or hepatitis in the current modification of the PGPS-induced arthritis model is inherent to the use of PGPS rather than the extension of periods in between reactivation. To what extent the hepatitis and splenitis resulted in adverse effects on general health is not known, since it may not always be easy to distinguish pain behavior from general malaise, although no clinical signs typical of *streptococcus* infection were observed. In addition to these systemic effects, variable mild polyarthritis of other joints (contralateral knee, occasional swelling of ankles) as reflected by low-grade inflammation was present. Slight hypersensitivity of the contralateral knee joint was also observed, although the by histology synovitis of those knee joints was shown to be very mild. To what extent the reactivation responses were exactly the same as in the short version of the model, is unclear. The memory response may be different upon reactivation after 4 compared to 2 weeks. Although most likely this is the case, the direction of the effect of this extension is unpredictable. Within a similar time frame, the effect of boost timing on the CD8+ T-cell memory response was shown to be dependent on the antigen itself, the antigen dose and actual exposure time [26,27], leading to enhancement of clonal expansion or to contraction [26]. This has not been investigated for this model and antigen. However, we did not see a clear difference in the magnitude of effects of reactivations compared to previous publications, although the pain parameters measured were not identical, precluding a direct comparison. In order to reduce variation in arthritis patterns and animal loss in the modified—or original—PGPS model, lower dosages of rhamnose or other adaptations, such as intra-articular rather than systemic reactivations, should be explored in order to comply with the 3Rs of experimental animal use [28]. An ideal animal model in general shows reproducible disease symptoms and progression, is relatively inexpensive, and displays a large enough effect to detect differences in the follow-up time. In this respect, the current modified arthritis model was capable of detecting differences between two therapeutic drug delivery systems, underscoring its potential as animal model of arthritis for long-term testing of DDSs. Indeed, the inhibition of joint swelling in the current arthritis model by TAA-loaded PLGA microspheres appeared to be lost after 32 days, while the PEA platform continued to inhibit joint swelling. This important difference between drug delivery platforms would not have been observed in the original version of this arthritis model [11,12]. Therefore, the modified arthritis model is well suited to test DDS for an extended follow-up period with relatively small group sizes. Notably, statistical analysis was done over the treatment period, and hence only delineated differences over that timeframe, instead of differences at the individual time points. Nonetheless, the course of the effects on pain and inflammation do suggest there might be differences in long-term therapeutic effectiveness between the two platforms.

Surprisingly, animals injected with the TAA-loaded microsphere platforms initially lost weight after injection, suggesting systemic exposure at bioactive levels. This may be explained by the release profiles; in vitro, we also observed an initial TAA burst from both microsphere platforms. Initial burst release is a known phenomenon for microparticle drug delivery, and is related to the presence of drug close to the surface and the high surface to volume ratio of the microparticles. It should be noted that the “burst” release still only delivers about 20% if the amount of the bolus corticosteroid injection applied as standard of care in clinic. TAA-loaded PLGA microsphere-treated animals recovered in body weight to the levels of untreated animal levels 15 days after injection, while those treated with TAA-loaded PEA microspheres did not catch up in body weight, suggesting the extended presence of systemic threshold levels of the drug using the PEA platform. This was further corroborated by the reduced incidence of hepatitis in PEA-TAA treated animals compared to untreated or PLGA-TAA treated rats. 

Differences in the effects between the PLGA and PEA platforms might be explained by their different degradation and release characteristics, leading to different TAA release levels; PLGA microspheres degrade via hydrolytic bulk erosion processes, whereas PEA microsphere degradation is mainly driven by enzyme activity [29]. Previous studies have shown degradation rates of PLGA drug delivery systems in vivo of on average 21 days [30,31]. Degradation of PEA drug delivery systems have been reported to take over 84 days [10,29,32]. The PLGA bulk erosion is reflected in the second burst of TAA release observed in vitro. The different degradation and release properties were further reflected in the temporal pattern of therapeutic effects, although this timing in vivo does not correspond to the in vitro burst release. Here, a clear loss of anti-inflammatory effect was noted for joints treated with TAA-loaded PLGA microspheres, where swelling recurred 32 days post microsphere injection. In vitro and in vivo release kinetics are seldom similar. TAA release from PLGA microspheres has been described to be faster in vivo than in vitro, with a full in vivo release after just 21 days, in contrast to the 35 day period found in vitro [31]. TAA release from PEA microspheres is more difficult to predict, considering that drug release from PEA microspheres is dependent on enzyme activity rather than hydrolysis, while enzyme activity will also be partially dependent on TAA-mediated suppression of inflammation [10]. The extended duration of the anti-inflammatory effects suggest that release in any case was longer that release by the PLGA platform. The difference in microsphere batches will have contributed relatively little to this phenomenon, given the consistent manufacturing procedures and hence properties of the loaded microspheres. In any case, a sufficient drug dose appeared to be available to successfully reduce joint swelling throughout the 84 days. This long-lasting inhibition of inflammation was confirmed by the very mild synovitis found by histopathology of joints treated with TAA-loaded PEA microspheres at the end of the study period. However, within the limitations of the group size of the present study, the attenuation of the pain induced changes in weight bearing eventually was lost for TAA delivery of both platforms, without any clear difference between the two platforms. In contrast, referred hyperalgesia was inhibited throughout the complete study period for TAA-loaded PEA microspheres. These processes are differently organized in the spinal and sensory neurons [33]. In that regard, referred hyperalgesia often only occurs with severe inflammation, whilst weight bearing asymmetries may already occur with lower grade inflammation. Considering that glucocorticoids act through three different primary mechanisms [34], the spatiotemporal drug concentration may have to overcome a certain threshold to elicit a therapeutic effect, which may be different between the different pain parameters. For example, synovial fluid TAA concentrations correlate with treatment efficacy in arthritis patients [35]. Possibly, TAA released by PEA microspheres was able to maintain local drug concentrations overcoming the pain thresholds for evoked- and non-evoked pain behaviors, in contrast to concentrations of TAA released by PLGA microspheres which only reduced the non-evoked pain behavior. The indication of therapeutic differences between the two platforms is also reflected in the different polarization. TAA released from PEA microspheres was able to shift the macrophage population towards an anti-inflammatory macrophage phenotype in the synovial tissue, compared to untreated synovial tissue or treated with TAA released from PLGA microspheres. Although we did not use a general macrophage staining, iNOS positivity in the synovial tissue usually represents macrophages as they are the predominant iNOS positive cell within the synovium, whereas T cells, B cells, and neutrophils are iNOS negative [36]. The mannose macrophage receptor (MMR) was used to quantify CD206-positive macrophages in using serial synovial tissue sections in addition to the iNOS immunohistochemistry. Therefore, the ratio of iNOS/cd206 positive cells in the subsequent synovial section probably represent the M1/M2 macrophage ratio in vivo. 

## 5. Conclusions

In conclusion, the current modified model of acute arthritis has good applicability for long-term testing of drug delivery systems but, due to its systemic inflammatory load, requires further optimization. Despite relative small group sizes, differences in effectiveness of TAA delivery between the PLGA and PEA microsphere platforms were clearly demonstrated. 

## Figures and Tables

**Table 1:** Incidence of granulomatous hepatitis or granulomatous splenitis.

Abnormality	No treatment	%	PLGA TAA	%	PEA TAA	%
granulomatous hepatitis	no–slight reaction (2/6)	**33**	no–slight reaction (3/6)	**50**	no–slight reaction (5/6)	**83**
	moderate reaction (1/6)	**17**	moderate reaction (1/6)	**17**		
	severe reaction (3/6)	**50**	severe reaction (2/6)	**33**	severe reaction (1/6)	**17**
granulomatous splenitis	no–slight reaction (2/6)	**33**			no–slight reaction (2/6)	**33**
	severe reaction (4/6)	**67**	severe reaction (6/6)	**100**	severe reaction (4/6)	**67**

## Supplementary Materials

The following are available online at http://www.mdpi.com/1999-4923/11/2/70/s1, Figure S1. Cartilage degeneration of affected knee joint (left panel) and contralateral knee joint (right panel) was quantified by Mankin scoring of knee joints treated with a single intra-articular injection of TAA-loaded PLGA microspheres (red) or TAA-loaded PEA microspheres (blue) and compared with untreated knee joints (no treatment). Data are presented as mean ± SD.
