# Supplementary material for: Applicability of a Modified Rat Model of Acute Arthritis for Long-Term Testing of Drug Delivery Systems
Source: Pharmaceutics. 2019 Feb 7;11(2):70. doi: 10.3390/pharmaceutics11020070 (PMC6409650; doi:10.3390/pharmaceutics11020070)
Supplement: Supplementary file 1 [file pharmaceutics-11-00070-s001.pdf]

pharmaceutics

Supplementary Materials:

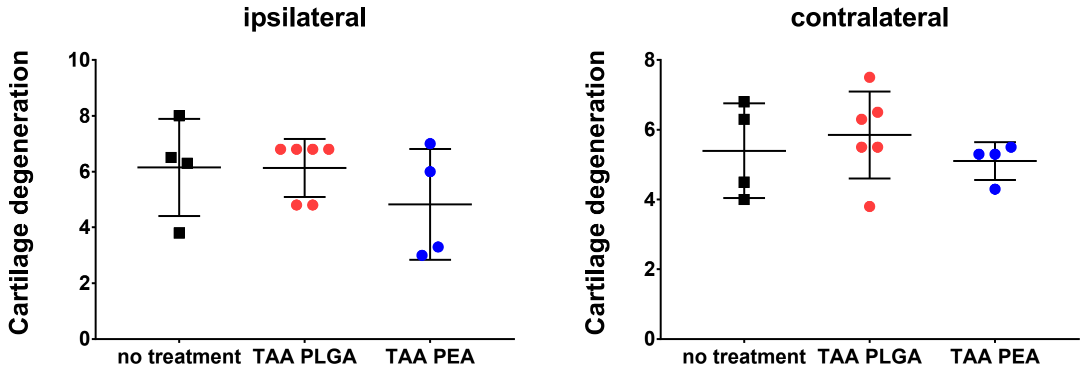

Figure S1. Cartilage degeneration of affected knee joint (left panel) and contralateral knee joint (right panel) was quantified by Mankin scoring of knee joints treated with a single intra-articular injection of TAA-loaded PLGA microspheres (red) or TAA-loaded PEA microspheres (blue) and compared with untreated knee joints (no treatment). Data are presented as mean ± SD.
